# Mode of Action Classifications in the EnviroTox Database: Development and Implementation of a Consensus MOA Classification

**DOI:** 10.1002/etc.4531

**Published:** 2019-09-05

**Authors:** Aude Kienzler, Kristin A. Connors, Mark Bonnell, Mace G. Barron, Amy Beasley, Cristina G. Inglis, Teresa J. Norberg‐King, Todd Martin, Hans Sanderson, Nathalie Vallotton, Peter Wilson, Michelle R. Embry

**Affiliations:** ^1^ European Commission, Joint Research Centre, Ispra Italy; ^2^ The Procter & Gamble Company, Cincinnati Ohio USA; ^3^ Environment and Climate Change Canada, Gatineau Quebec Canada; ^4^ Gulf Ecology Division US Environmental Protection Agency, Gulf Breeze Florida; ^5^ Dow Chemical Company Midland Michigan USA; ^6^ US Environmental Protection Agency Duluth Minnesota; ^7^ US Environmental Protection Agency, Cinncinati Ohio; ^8^ Aarhus University Aarhus Denmark; ^9^ Dow Chemical Company Basel Switzerland; ^10^ Sanofi, Bridgewater New Jersey USA; ^11^ Health and Environmental Sciences Institute Washington DC

**Keywords:** Mode of action, aquatic toxicity, classifications, EnviroTox database, ecological risk assessment

## Abstract

Multiple mode of action (MOA) frameworks have been developed in aquatic ecotoxicology, mainly based on fish toxicity. These frameworks provide information on a key determinant of chemical toxicity, but the MOA categories and level of specificity remain unique to each of the classification schemes. The present study aimed to develop a consensus MOA assignment within EnviroTox, a curated in vivo aquatic toxicity database, based on the following MOA classification schemes: Verhaar (modified) framework, Assessment Tool for Evaluating Risk, Toxicity Estimation Software Tool, and OASIS. The MOA classifications from each scheme were first collapsed into one of 3 categories: non–specifically acting (i.e., narcosis), specifically acting, or nonclassifiable. Consensus rules were developed based on the degree of concordance among the 4 individual MOA classifications to attribute a consensus MOA to each chemical. A confidence rank was also assigned to the consensus MOA classification based on the degree of consensus. Overall, 40% of the chemicals were classified as narcotics, 17% as specifically acting, and 43% as unclassified. Sixty percent of chemicals had a medium to high consensus MOA assignment. When compared to empirical acute toxicity data, the general trend of specifically acting chemicals being more toxic is clearly observed for both fish and invertebrates but not for algae. EnviroTox is the first approach to establishing a high‐level consensus across 4 computationally and structurally distinct MOA classification schemes. This consensus MOA classification provides both a transparent understanding of the variation between MOA classification schemes and an added certainty of the MOA assignment. In terms of regulatory relevance, a reliable understanding of MOA can provide information that can be useful for the prioritization (ranking) and risk assessment of chemicals. *Environ Toxicol Chem* 2019;38:2294–2304. © 2019 The Authors. *Environmental Toxicology and Chemistry* published by Wiley Periodicals, Inc. on behalf of SETAC.

## INTRODUCTION

In the last decade, there has been a shift in the risk‐assessment paradigm toward an improved understanding of toxicological mechanisms (Krewski et al. 2010). This improved mechanistic understanding would reduce reliance on traditional animal testing by allowing chemical grouping to increase prioritization and assessment efficiency, improve toxicity extrapolations, and increase the potential use of animal alternative methods, which is a long‐term policy goal of most chemical management programs. Mode of action (MOA) can be defined as a functional change at the cellular level, in contrast to the mechanism of action or molecular initiating event. It is important in classifying chemicals because it represents an intermediate level of complexity between molecular mechanisms and physiological or organismal outcomes and provides an organizing scheme for chemical classification (Carriger et al. 2016; Kienzler et al. 2017). The knowledge of MOA is fundamental in aquatic environmental risk assessment, where the ultimate goal is to protect the communities and ecosystems, because different species or taxa might have different sensitivity to a particular MOA. Thus, it might help to identify the most sensitive trophic level for a given chemical. Several MOA frameworks have been developed in aquatic ecotoxicology, mainly based on acute fish toxicity (Kienzler et al. 2017): the Verhaar classification framework (Verhaar et al. 1992), further modified by Enoch et al. (2008); the US Environmental Protection Agency (USEPA) Assessment Tool for Evaluating Risk (ASTER) quantitative structure–activity relationship (QSAR) application (Russom et al. 1997; US Environmental Protection Agency 2012); the OASIS MOA, originally developed for the 3‐dimensional chemistry‐capable Tissue Metabolism Simulator (TIMES) acute toxicity software by the Laboratory of Mathematical Chemistry (LMC), based on Dimitrov et al. (2003); and the USEPA's Mode of Action and Toxicity (MOATox) database (Barron et al. 2015). The latter database has been used to further develop the Toxicity Estimation Software Tool (TEST; US Environmental Protection Agency 2016), which is able to predict the MOATox MOA according to linear discriminant models (Martin et al. 2013, 2015).

The definitions of MOA used are unique to each of the classification schemes investigated. They have been previously described and critically compared (Kienzler et al. 2017), and it has been shown that discrepancies exist between those different frameworks. Because the various approaches have been built on different sets of rules, use different knowledge bases, and lack harmonization, they can give contradictory results. As a result, although the existing MOA frameworks have great potential to improve risk assessment and strengthen the use of alternative methods, a reliable tool for classification of an MOA assignment still eludes ecotoxicology.

Research to develop screening and early tier methods to more easily classify or assess chemicals using approaches such as the ecological threshold of toxicological concern (eco‐TTC; Belanger et al. 2015) is ongoing. In this context, a database (EnviroTox) has been built with more than 91 000 curated records of ecotoxicological data (Connors et al. 2019). An accompanying tool facilitates derivation of threshold concentration‐based points of departure (e.g., median effective concentration and median lethal concentration [LC50]) either from an ecotoxicological data distribution or on a predicted‐no‐effect concentration distribution based on the underlying, user‐filtered data set. The database also includes information on each chemical such as its physicochemical properties or its MOA classification according to the Verhaar (modified) framework, ASTER 2.0, the USEPA's TEST 4.2, and LMC OASIS schemes.

Given that Kienzler et al. (2017) noted the discrepancies between various MOA classification approaches, the objective of the present study was to critically evaluate the EnviroTox data set with the aim of seeking a consensus MOA assignment from the currently available classification schemes. This consensus MOA would enhance the robustness of the database and provide confidence in the classification of chemicals by MOA for threshold concentration derivation. The MOA is also needed to select appropriate critical body residues for determining internal toxicity thresholds (McCarty and Mackay 1993; Escher et al. 2011) and can inform the appropriate calculation for cumulative chemical effects assessment.

In the present study, we have proposed a set of consensus rules based on a 4‐model MOA comparison undertaken for approximately 3900 organic substances in the EnviroTox database. A consensus was sought so that binary decisions could be made to classify the 3900 organic substances as non–specifically acting (i.e., narcotic), specifically acting, or unclassifiable. This binary classification was chosen for 2 main reasons: first, the tools to predict specific MOAs are generally not aligned, which makes it complicated to go into deeper detail; and second, those tools taken individually are not robust enough to confidently classify into specific MOA bins. In addition, a confidence rank was assigned to each MOA designation based on the degree of model consensus. This provides users of the EnviroTox database a transparent understanding of the MOA classification by chemicals and the related uncertainty. This understanding of MOA, even at a simple binary level, helps to identify chemicals that could be of concern in a risk‐assessment context, and this information can be used for the prioritization (ranking) and risk assessment (understanding of interspecies differences in sensitivity) of chemicals in a regulatory context. Because specifically acting substances are more potent than non–specifically acting substances and the specific MOA can more directly target a particular species, differences in species sensitivity tend to be higher for substances with a specific MOA than for narcotics. The consensus approach used in the present study is conceptually similar in part to the MOA descriptor used in the hazard profile contained in the Ecological Risk Classification Approach developed by Environment and Climate Change Canada or the reprioritization of 640 organic substances on the Domestic Substances List for the third phase of the Chemicals Management Plan (Environment and Climate Change Canada 2016; Organisation for Economic Co‐operation and Development 2018).

The present study has 3 overall objectives: 1) to develop a consensus approach to MOA classification that can harmonize across assignment schemes, 2) to assess and ensure an accurate MOA assignment in the EnviroTox data to enhance the quality and utility of the database, and 3) to assess and discuss the distribution of MOAs represented in the large database.

## MATERIALS AND METHODS

### Database

The EnviroTox database (Health and Environmental Sciences Institute 2018; Connors et al. 2019) was originally created to support the development of the eco‐TTC (Belanger et al. 2015). The database was constructed to contain curated in vivo aquatic toxicity results relevant for ecological risk assessments. A large and diverse database was amassed from a variety of public and private sources, encompassing a wide range of chemistries, species, and regulation‐relevant endpoints. The Stepwise Information‐Filtering Tool (SIFT) was used to provide an objective framework for data curation (Beasley et al. 2015) and was applied to the approximately 220 000 initial records, resulting in a final curated database of 91 217 records. Considerations of relevant trophic levels, experimental duration, inclusion of specific effect measurements, and reported test statistics were taken into account. Duplicate entries and extreme outliers were also flagged and removed. A detailed account of the curation steps can be found in the companion database article (Connors et al. 2019) and within the database and tool user's guide (Health and Environmental Sciences Institute 2018).

Additional curation efforts were applied to systematically validate the Chemical Abstracts Service (CAS) number, chemical name, and simplified molecular‐input line‐entry system notation (SMILES). Briefly, all chemical CAS numbers were run through the USEPA's Chemistry Dashboard (comptox.epa.gov/dashboard). If a chemical CAS and chemical name match was found, the chemical was considered “validated,” and the SMILES was extracted. If a match was not found, additional databases and tools (e.g., SciFinder) were used to identify and validate the chemical. If no validation could be made, the chemical was excluded from the database.

The EnviroTox database count is 91 217 records and includes 1563 species and 4016 unique CAS numbers. Compounds containing a heavy metal (metallic element with density >5) were identified based on SMILES notation. This included a total of 317 unique CAS numbers and corresponds to approximately 19% of the total EnviroTox database records. Because the toxicity of some metal‐containing compounds may be driven by the presence of the freely dissolved metal ion, a total of 140 metal‐containing compounds were collapsed into 24 different “dummy metal ion CAS numbers.” This lowers the number of unique CAS numbers in the database to 3900. These compounds have no modeled data, including MOA classifications. In addition, metals are often out of domain for MOA classification schemes. In this case, a model will list the MOA as “unknown,” “not classifiable,” or “out of domain.”

### MOA assignments

To allow grouping of chemicals based on the MOA, 4 structure‐based MOA classification approaches were applied to each chemical: Verhaar (modified), TEST 4.2, OASIS, and ASTER 2.0. This expands on earlier work performed by Kienzler et al. (2017) on a previous version of the database. The CAS number and SMILES were used as an input in the Organisation for Economic Co‐operation and Development (OECD) QSAR Toolbox, Ver 4.2, to obtain the Verhaar (modified) and OASIS results, ensuring that the input SMILES was the SMILES used for profiling of MOA (i.e., not the CAS number assigned by the toolbox). In addition, the USEPA's new chemical and Ecological Structure Activity Relationships (ECOSAR) chemical categories have been included to provide information on structure‐based grouping and were also extracted via the OECD QSAR Toolbox using the appropriate chemical profilers. When a prediction cannot be obtained using these approaches, it indicates that a chemical is outside of the structural domain of application. In general, the domain of application is not well documented in any of the 4 approaches (i.e., a prediction of MOA can be achieved or not, but the degree of structural coverage in model training sets is not provided, to allow judgment of domain applicability; the exception is implementation of the OASIS MOA in the TIMES suite of models, where a quantitative 3‐level domain of application is provided).

The ASTER and TEST classifications were determined by running the corresponding USEPA acute fish toxicity models that provide toxicity predictions according to MOA determinations. To obtain ASTER classifications, the SMILES corresponding to the listed CAS numbers were first processed through the proprietary ASTER tool. If the SMILES was not present or returned an error, then the corresponding CAS number was submitted to maximize the number of CAS numbers given an ASTER assignment. When the SMILES represented a structure described as “impossible,” ASTER returned an error message describing the structure discrepancies, and no MOA was assigned. When the SMILES represented a mixture, no MOA was assigned because mixtures are outside the ASTER applicability domain. When no match was found from the SMILES or CAS number, no MOA assignment was returned.

Predictions of MOA were obtained using TEST Ver 4.2 (US Environmental Protection Agency 2016), which determines MOA based on the MOATox classification scheme (Barron et al. 2015). First, TEST assigns theoretical molecular descriptors based on chemical structure; then, it uses a linear discriminant analysis model to determine the MOA (Martin et al. 2013, 2015).

The OASIS MOA results, obtained using the OECD QSAR Toolbox Ver 4.2, were derived from the toolbox profiler “Acute Aquatic Toxicity MOA by OASIS.” This MOA approach, as implemented in the OECD QSAR Toolbox, is conceptually similar to the Verhaar scheme and thus uses a decision‐rule approach derived from its own knowledge base (chemical training set). The OASIS MOA has been implemented in the OECD QSAR Toolbox since Ver 3.4.

### MOA consensus binning and confidence scoring

To simplify the grouping of chemicals by MOA and to strengthen the confidence we can have in such a grouping, each chemical was assigned to one of 3 “general” groupings based on the degree of consensus between the evaluated schemes and the concordance between schemes shown in Table 1: narcotic (N), specifically acting (S), or unclassified (U). Briefly, the N bin brings together structural categories of narcotic chemicals, including nonpolar, polar, and ester narcotics. The S bin contains all reactive chemicals, which theoretically are expected to elicit a toxic response at the site of toxic action (specific or not) at lower concentrations than narcotic chemicals, whereas the U bin contains the unclassified chemicals.

**Table 1:** Mode of action classification table and concordance “bins”

Verhaar		ASTER		TEST		OASIS	
Classification	Bin	Classification	Bin	Classification	Bin	Classification	Bin
Class 1 (narcosis or baseline toxicity)	N	Nonpolar narcosis	N	Narcosis	N	Base surface narcotics	N
Class 2 (less inert compounds)		Polar narcosis				Narcotic amines	
		Ester narcosis				Phenols and anilines	
						Alpha, beta‐unsaturated alcohols	
						Esters	
Class 3 (unspecific reactivity)	S	Diester toxicity	S	Reactivity	S	Reactive unspecified	S
Class 4 (compounds and groups of compounds acting by a specific mechanism)		Reactive		Neurotoxicity		Aldehyde	
		Chloro‐diester‐based reactivity		AChE inhibition			
		Carbonyl (C=0)–based reactivity		Electron transport inhibition			
		Carbonyl reactivity		Iono/osmoregulatory/circulatory impairment			
		Alkylation/arylation‐based reactivity					
		Acylation‐based reactivity					
		Sulfhydryl (‐S‐H)–based reactivity					
		Reactive dinitroaromatic group					
		Nitroso‐based reactivity					
		Quinoline reactivity					
		Acetamidophenol reactivity					
		Reactive diketones					
		Acrylate toxicity					
		N‐halogenated acetophenone inhibition					
		Hydrazine‐based reactivity					
		Isocyanate (‐N=C=O)–based reactivity					
		Pyridinium compounds					
		Neurotoxicant: DDT‐type					
		Neurotoxicant: pyrethroid					
		Neurotoxicant: cyclodiene‐type					
		Neurotoxicant: strychnine					
		Neurotoxicant: nicotine					
		Organophosphate‐mediated AChE inhibition					
		Carbamate‐mediated AChE inhibition					
		Uncoupler of oxidative phosphorylation					
		Respiratory blocker: azides and cyanides					
Class 5 (not classifiable)	U	Unknown mode of action or out of domain	U	Unknown or out of domain	U	Unknown or out of domain	U

AChE = acetylcholine esterase; ASTER = Assessment Tool for Evaluating Risk; N = narcotic; S = specifically acting; TEST = Toxicity Estimation Software Tool; U = unclassified.

Each MOA scheme assignment was collapsed into one of the 3 bins as assigned in Table 1. A 4‐letter code, corresponding to the TEST, ASTER, OASIS, and Verhaar bins, respectively, was then assigned to each chemical. Based on this 4‐letter code, a consensus MOA was determined for each chemical, and a confidence score was assigned based on the following rules. All 4 schemes in agreement (e.g., NNNN, SSSS, UUUU): confidence score of 3. Three schemes in agreement (e.g., NNNS, SSNS): confidence score of 2. Two schemes in agreement with the other 2 as “U” (e.g., NNUU, SUSU): confidence score of 1 and assignment made on the non‐“U” assignment. All other combinations: assigned a consensus MOA of “U” and a confidence score of 0. A full description of the possible 4‐letter codes and their related confidence scoring is provided in Table 2.

**Table 2:** Summary of mode of action (MOA) assignments and confidence scores within the EnviroTox database

Consensus MOA	Confidence score	CAS no.	% of chemicals	No. of studies	% of studies	4‐letter code
N	3	454	11.6	8181	9.0	NNNN
N	2	890	22.8	15 903	17.4	NNNS, NNNU, UNNN, NNSN, SNNN, NSNN, NUNN
N	1	224	5.7	3027	3.3	NNUU, NUNU, UNNU, UUNN
S	3	179	4.6	10 783	11.8	SSSS
S	2	410	10.5	10 071	11.0	NSSS, SUSS, USSS, SSNS, SSSU, SNSS, SSSN
S	1	66	1.7	1295	1.4	USSU, UUSS, SUSU, SSUU
U	3	227	5.8	17 358	19.0	UUUU
U	2	208	5.3	3724	4.1	UUSU, UUNU, UNUU, USUU, NUUU, SUUU
U	0	1244	31.9	20 875	22.9	NUNS, NNSS, UNSS, UNSU, NUSU, SNSU, UNNS, NNSU, NSUU, SUNU, SNNU, NSSU, NSNS, NSSN, NSNU, USNU, UUNS, SNNS, SSNU, NUSS, SNSN, USSN, NUSN, SUNS, USNS, UNSN, UUSN, SNUU

CAS = Chemical Abstracts Service; N = narcotic; S = specifically acting; U = unclassified.

For the concordance analysis, it should be highlighted that the OASIS classification refers to chemical groups that might not always be related to a unique MOA (in this sense, it represents a diverse structure–activity relationship). That is the case, for example, with phenols: simple phenols will have a polar narcotic MOA, but they can also have a particular reactivity or specific MOA, such as an uncoupler of oxidative phosphorylation, if they contain an aromatic nitro group. Those phenols with an aromatic nitro group will most probably be classified in the S bin in the Verhaar framework, ASTER, and MOATox but will be classified in the N bin by OASIS. They would then ultimately be given an S classification, but the N classification by OASIS would decrease the confidence score. As another example, in ASTER, ester narcosis corresponds to chemicals with an ester structure that do not contain chlorodiesters, acrylates, or diesters (in which case they would fall under reactivity/ester), whereas all the esters would be identified as N in OASIS.

## RESULTS AND DISCUSSION

### Distribution of MOA assignments

**Figure 1:**
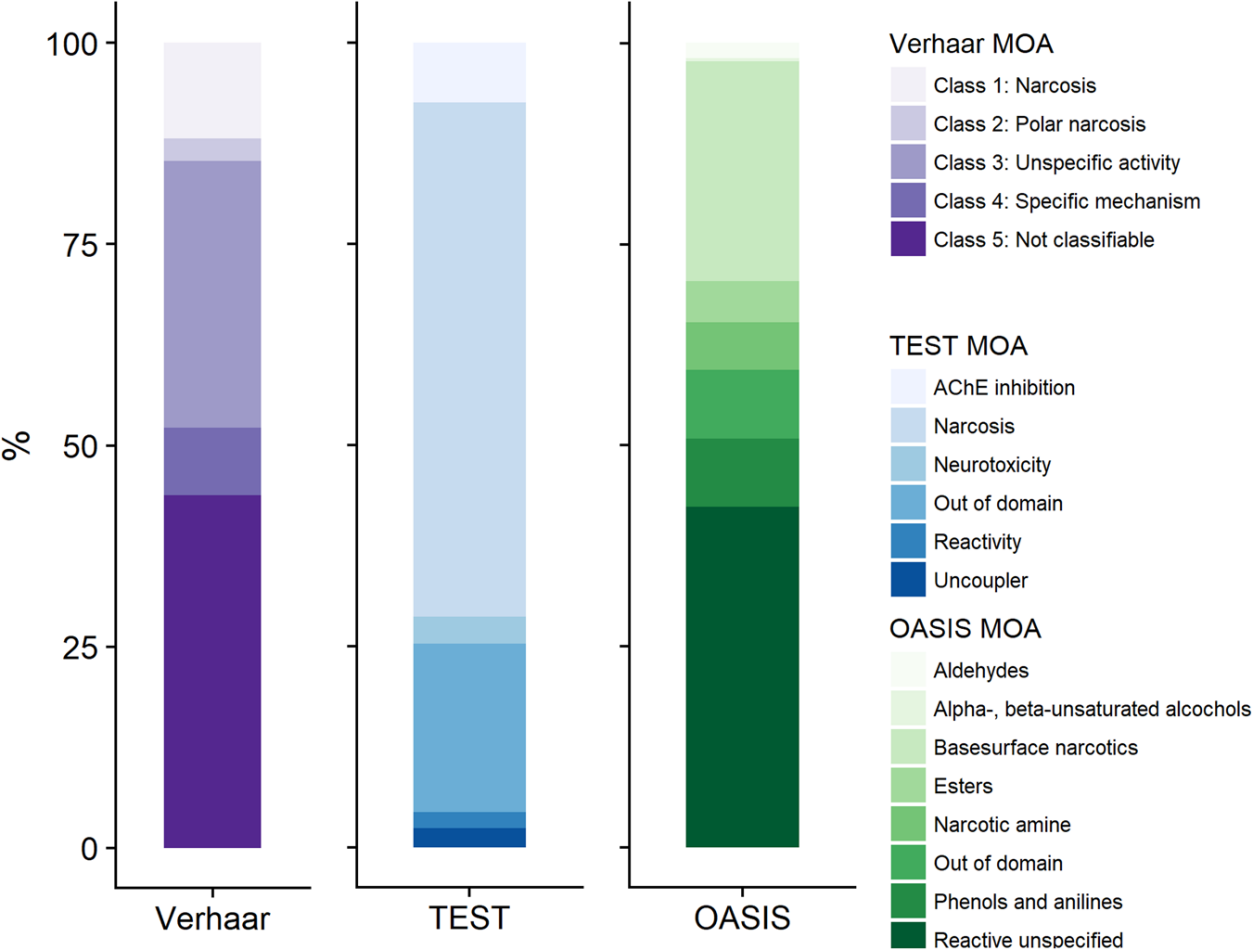
Percentage of chemicals within the EnviroTox database assigned to each mode of action (MOA) for Verhaar, Toxicity Estimation Software Tool, and OASIS. The Assessment Tool for Evaluating Risk classification is not represented because the graph was lacking clarity as a result of the high number of MOA classes; however, these data are available in Supplemental Data, Table S1. AChE = acetylcholinesterase; TEST = Toxicity Estimation Software Tool.

Within the Verhaar (modified) classification scheme, class 4 chemicals account for approximately 25% of the database entries (detailed in Supplemental Data, Table S1) but for only 8% of the chemicals (Figure 1). Those chemicals are therefore quite data‐rich when compared to the narcotics, which represent approximately 15% of the chemicals but only 10% of the database entries. Based on this scheme, a majority of the chemicals are classified as reactive (33% as unspecific reactivity and 8% as specifically acting chemicals) for only 15% of narcotics.

An inverse trend is observed for the ASTER and TEST classifications, as shown in Supplemental Data, Table S2. Within those 2 frameworks, 61 and 64% are classified as narcotics, respectively, whereas 17 and 15% are classified in the various reactive subclasses, respectively. As an example, in the TEST scheme, 7% of the chemicals in the database are classified as acetylcholinesterase inhibitors, 2% as reactive, 3% as neurotoxicants, and 2.4% as uncouplers (Supplemental Data, Table S1).

Regarding the OASIS classification scheme, 47% of the chemicals were classified in a chemical family that could be related to a narcotic MOA and 44% were classified as unspecific reactivity. Of the 3900 chemicals present in the database, 44% are not classifiable according to the Verhaar rules, whereas 22 and 21% are identified as out of domain by the ASTER and TEST classification tools, respectively. In the OASIS schemes, this percentage falls to 8% (Supplemental Data, Table S1).

### Summary of consensus MOA assignments and confidence scores

**Figure 2:**
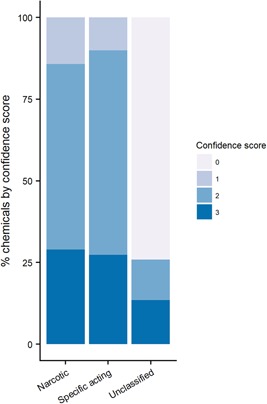
Confidence in the consensus narcotic, specifically acting, and unclassified designations. Consensus narcotic = 1568 chemicals; specifically acting = 655 chemicals; and unclassified = 1679 chemicals.

As shown in Table 2, the 4 MOA schemes agree for an N or S classification for only 16% of the chemicals present in the database, where the classification confidence is high (score = 3); this increases to 22% if we also consider the full agreement for U. For a further 38%, a confidence score of 2 shows that there is good agreement in the classification of narcosis, specifically acting, and unclassified across the individual models. Therefore, for 60% of the chemicals, the consensus MOA can be regarded as quite robust. For the remaining chemicals, the consensus classification and individual classifications are equivocal (i.e., no consensus) and should be regarded with caution. In addition, Figure 2 shows that 29% of the chemicals classified as narcotics (N) by the 4‐model consensus bin have a high confidence score (3) where all 4 schemes matched, whereas 57% of the narcotics have a medium confidence score (2). For chemicals classified as specifically acting (S) in the consensus MOA scheme, the trend is similar: 27% have a high confidence score (3), and 63% have a medium confidence score (2). Moreover, although there are fewer specifically acting chemicals than narcotic chemicals in the database (17 and 40%, respectively), these chemicals have disproportionately more data than the narcotic chemicals because they represent 24% of the studies (30% for the narcotics; Table 2). It has to be noted that a high proportion of those chemicals classified as U had a confidence score of 0 (i.e., there is no agreement between the 4 MOA schemes).

Sixteen percent of the total chemicals were given a consensus MOA of N or S with the highest confidence (3; Supplemental Data, Table S3). Approximately one‐third of the chemicals in the database (33%) were assigned an S or N consensus MOA with a moderate confidence score of 2, with 59% of these being classed as narcotics and 27% classed as specifically acting. Approximately 6% of the total chemicals were assigned unclassified (U) because of being out of domain of the models (confidence score = 3), and 37% had conflicting MOA assignments (confidence score = 2 or 0).

**Figure 3:**
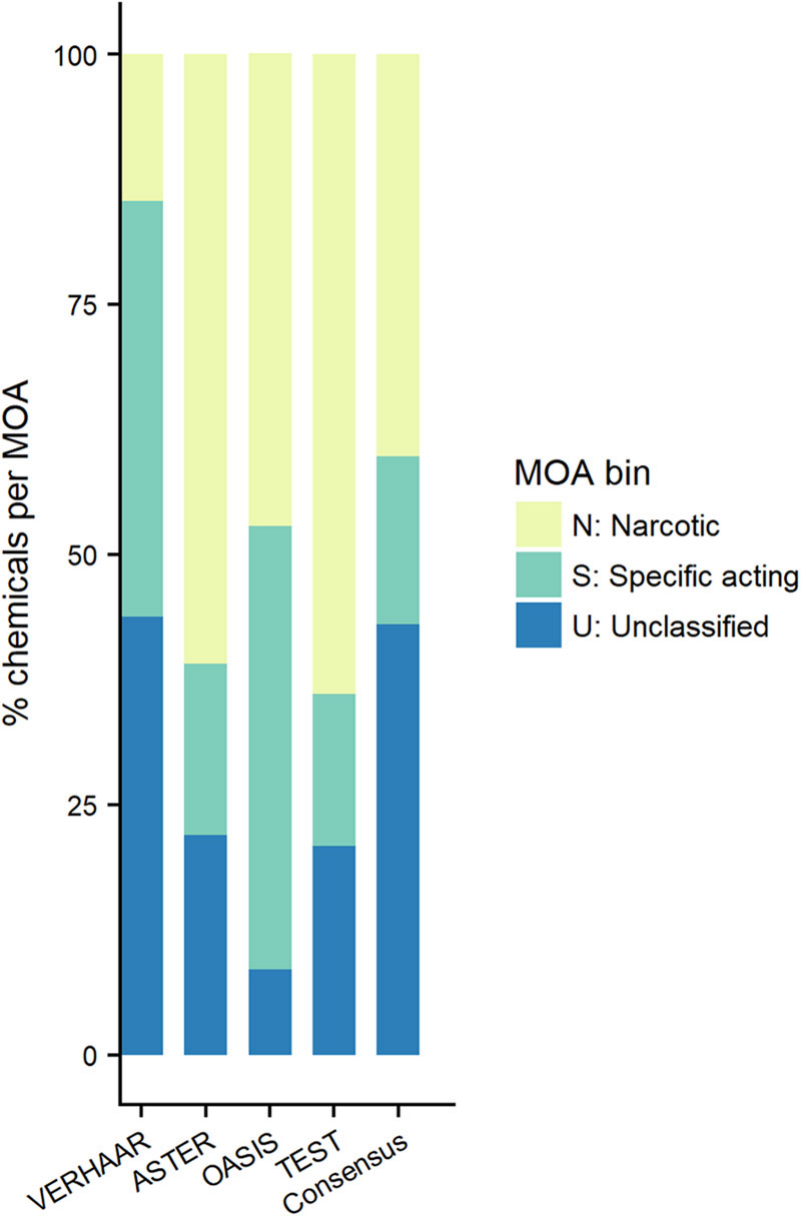
Percentage of chemicals that are classified into each mode of action (MOA) classification bin. ASTER = Assessment Tool for Evaluating Risk; TEST = Toxicity Estimation Software Tool.

Figure 3 compares the MOA (N, S, U) that would be attributed by each classification scheme individually to the consensus classification (Supplemental Data, Table S2). Both ASTER and TEST classify the majority of the chemicals (>60%) as narcotics, with approximately 15% as specifically acting and approximately 20% unclassified. Also, OASIS classified a large proportion of chemicals as narcotics (47%), but fewer chemicals were unclassified (9%) and a higher number was classified as specifically acting (44%). Conversely, the Verhaar classification scheme classified a much smaller proportion as narcotics (15%), but a much larger number were unclassified (>40%).

A significant portion of the EnviroTox database chemicals cannot be classified based on MOA (43%) for 2 primary reasons. First, the chemical may be out of the model domain of application (i.e., a significant amount of the chemical structure is not identified by the rules or structural domain of the MOA classification scheme used). Second, MOA classification cannot be assigned confidently. However, a conservative approach would be to assign a specifically acting designation to those chemicals where 2 models suggest narcosis and 2 models suggest specifically acting by assigning these as “reactive unspecified” (i.e., using the OASIS nomenclature). This would result in 10% becoming classified as specifically acting with a confidence score of 1. Ultimately however, for over half of the database (61%), there is relatively high confidence (i.e., score 3 or 2) in the selected MOA classification, as shown in Table 2.

### MOA assignments and effect values

**Figure 4:**
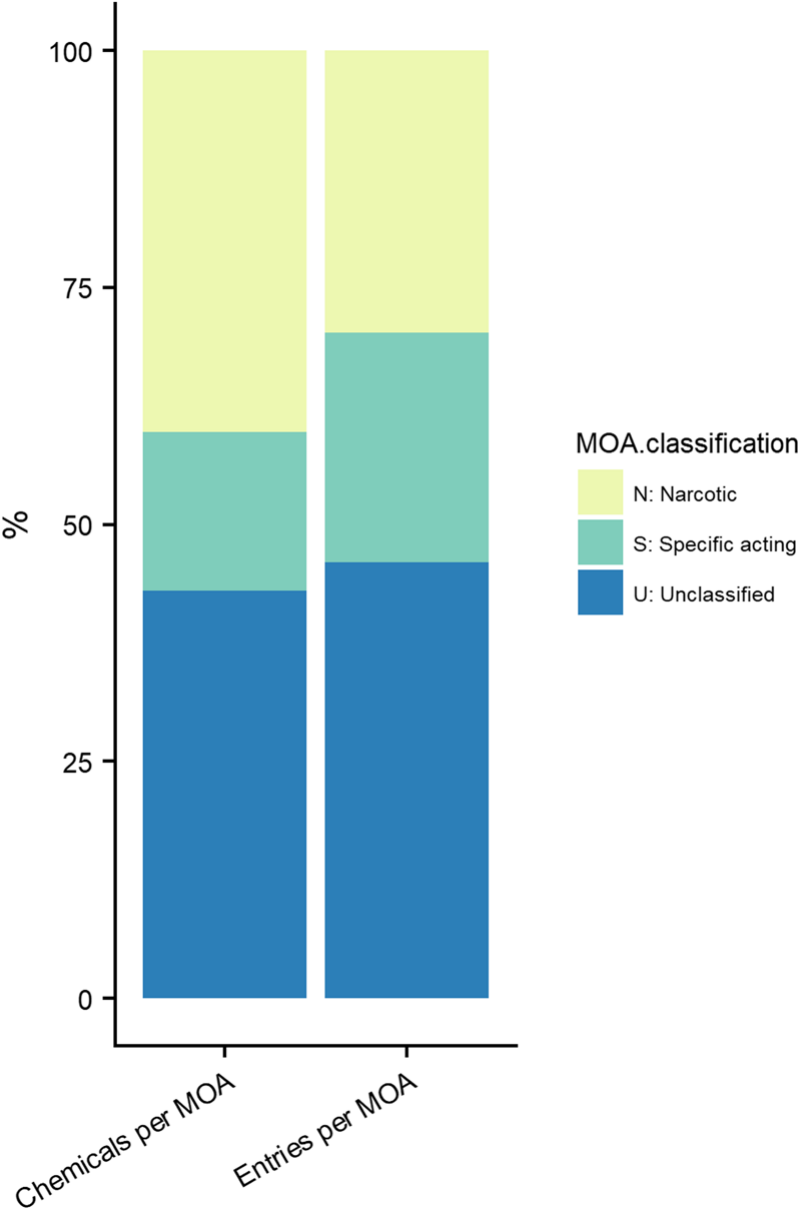
Chemical and data coverage within the consensus mode of action (MOA) bins for acute fish toxicity experiments.

Given that a specifically acting substance is expected to have a higher potency than a non–specifically acting substance, it is useful to compare assigned QSAR‐based MOAs to empirical acute toxicity data as a means to ground‐truth potency trends in the EnviroTox data set. However, trend analysis is sensitive to the bias of data availability. Figure 4 shows that there is 60% of the chemical space covered by acute fish toxicity data (i.e., 24‐ to 96‐h LC50) for narcotic and specifically acting chemicals when combined. A substantially higher proportion of chemicals are classified as narcotics within this space (Figure 4). There is also 60% study coverage when MOAs are combined (Figure 4). However, when MOA is viewed separately, studies on specifically acting substances make up a larger proportion of the total studies available for an assigned MOA. Uneven chemical and study space representation can introduce bias for statistical analysis if unweighted, and thus results are also presented using all data entries (i.e., reflects the current status of acute fish data in the EnviroTox database according to assigned MOA).

**Figure 5:**
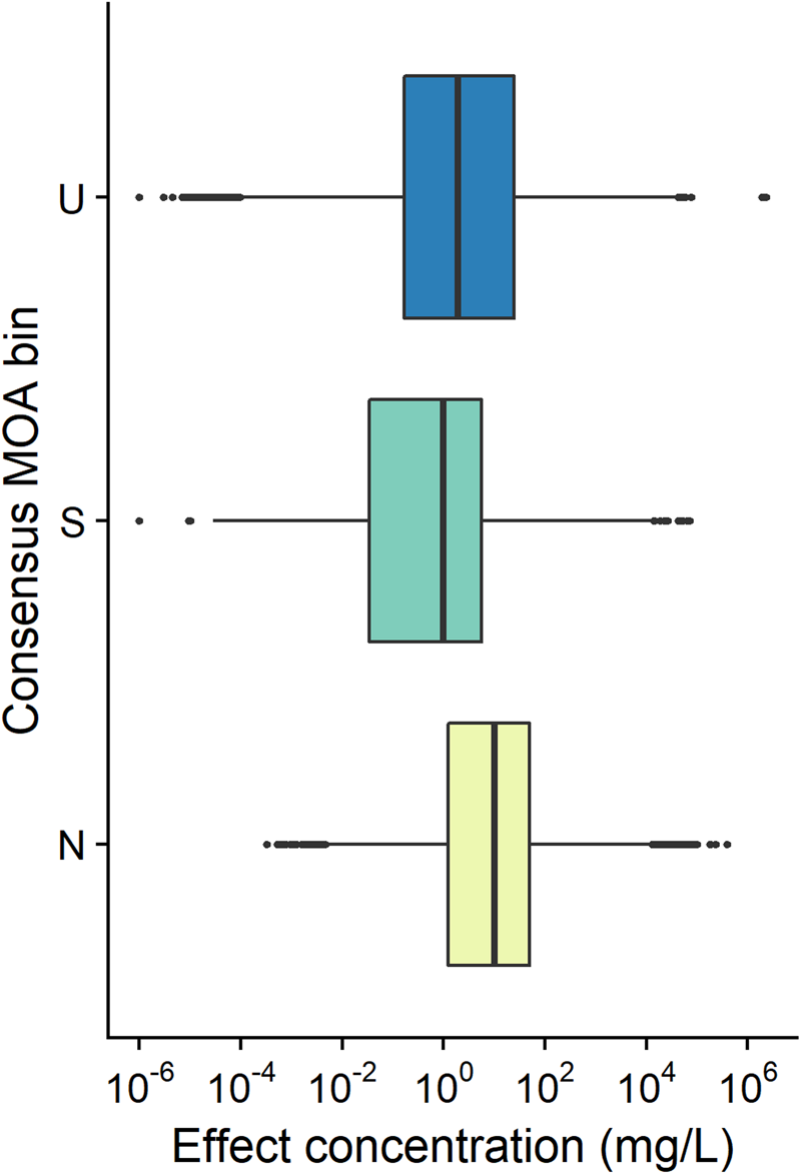
Distribution of acute fish effects for narcotic, specifically acting, and unclassified consensus mode of action (MOA) bins. N = narcotic; S = specifically acting; U = unclassified.

Figure 5 shows the distribution of acute toxicity data for fish for the 2817 chemicals of the database that have such data, for the different consensus MOAs. The complete range of effects is quite wide, from 10^−4^ to 10^6^ mg/L for narcotics and from 10^−6^ to 10^5^ for specifically acting chemicals. However, the majority of narcotic chemicals have an effect ranging from 10^0^ to 10^2^ mg/L, whereas this range is shifted to 10^−2^ to 10^1^ mg/L for the specifically acting chemicals. Thus, the increased toxicity of specifically acting chemicals is clearly seen, with the median of the distribution for narcotic chemicals at 10 mg/L and that for specifically acting chemicals at 1 mg/L.

**Figure 6:**
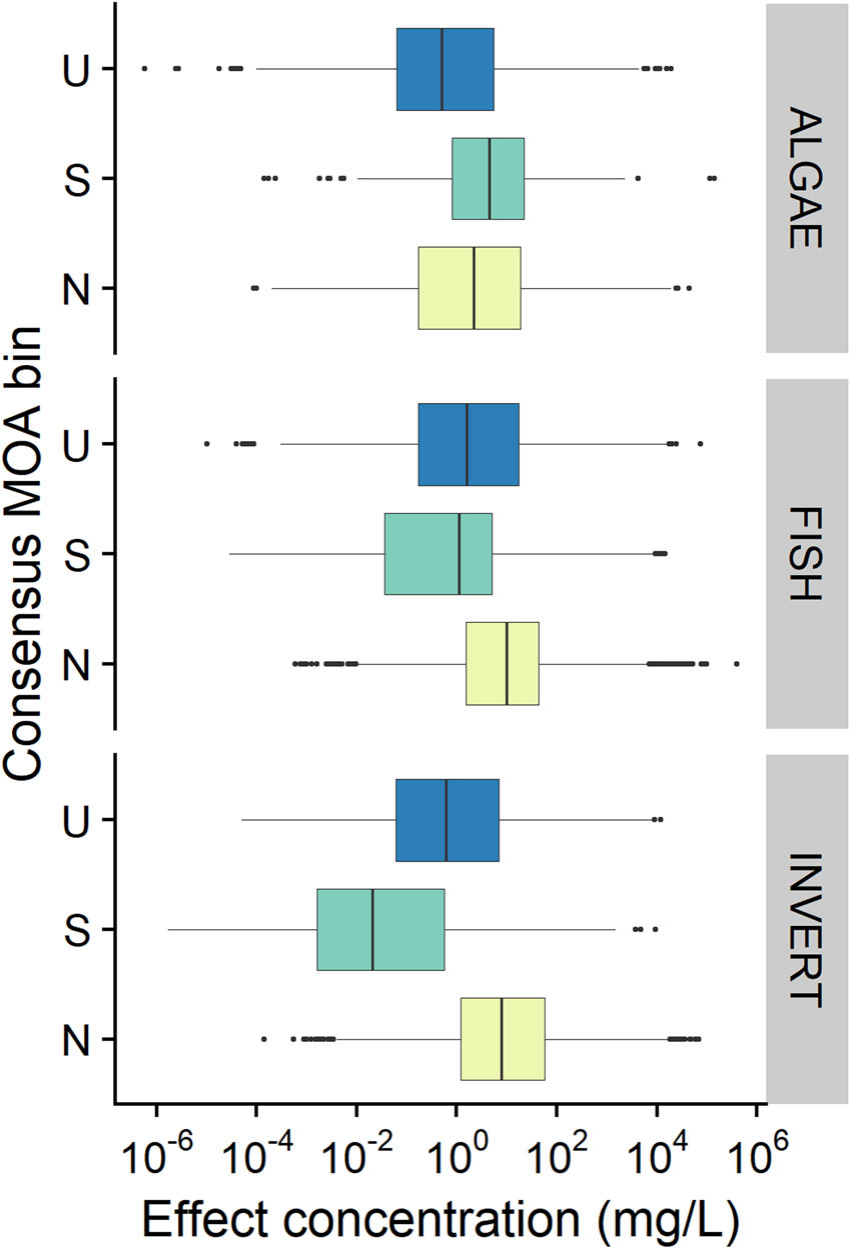
Distribution of acute effects for narcotic, specifically acting, and unclassified consensus mode of action (MOA) bins, by trophic level. *n* = 758 chemicals, with 5251, 33 056, and 15 603 studies for algae, fish, and invertebrates, respectively. N = narcotic; S = specifically acting; U = unclassified.

When considering the reduced data set of chemicals that have a complete acute data set (i.e., available acute toxicity data for algae, invertebrate, and fish, which is the case for 758 chemicals in the database), one can compare the distribution of effect by trophic level (Figure 6).

Figure 6 shows that, more particularly for fish and invertebrates, specifically acting chemicals are more toxic than narcotics. This is not surprising for fish (as previously shown in Figure 5) because most of the individual classification frameworks on which this consensus classification is based are built on acute fish toxicity. However, this trend is surprisingly even more pronounced for invertebrates, for which there is no overlap of the second and third quartiles of the effect value for narcotics and specifically acting chemicals. Thus, 50% of the acute toxicity data for invertebrates for those 758 chemicals ranges from 10^−3^ to <1 mg/L for specifically acting chemicals, whereas it ranges from 1 to 10^2^ mg/L for narcotic chemicals, with the median for N and S chemicals being quite distinct (nearly 3 orders of magnitude). For fish, the distinction is less clear, with a similar range of effects for narcotic chemicals as for invertebrates but with an effect range for 50% of the value from approximately 10^−2^ to 10 mg/L for S chemicals. The median between N and S chemicals is separated by 1 order of magnitude only for fish. For algae, the range of effect of N and S chemicals nearly overlaps, with N chemicals even appearing to be more toxic.

Thus, the data support the general trend of specifically acting chemicals being more toxic for both fish and invertebrates and further demonstrate that these MOA assignment schemes may not be appropriate for algae. Algae can be considered a sentinel taxon for chemicals with the toxic action of narcosis (perturbation of the cell membrane). Membrane–water partitioning, or *K*_MW_ (Bitterman and Goss 2017), thus becomes an important predictor for algal toxicity from water‐borne exposures and particularly for ionic chemicals such as cationics. Given that algae have much lower xenobiotic metabolic competence compared with fish, metabolism can largely be disregarded as a major toxicokinetic factor influencing toxicodynamics in algae. It is therefore hypothesized that the overlap in toxicity values of narcotic and specifically acting chemicals may be driven by similarity in *K*_MW_, which can ultimately exceed the critical membrane concentration and result in cell death (Bittermann and Goss 2017).

In addition, these data show the pitfalls of using media‐based (as opposed to internal) chemical concentrations for MOA comparisons. Toxicity values based on aqueous concentrations can be influenced by chemical thermodynamics (e.g., maximum solubility in water) and toxicokinetics (uptake, distribution, metabolism, and elimination). For example, the concentration of acute median lethality measured in water for narcotic chemicals ranges 5 orders of magnitude versus less than 1 order of magnitude based on internal residues (Mackay et al. 2014). Both of these factors influence the concentration at the site of toxic action in an organism, but because traditional ecotoxicology has focused on measurement of the exposure medium, there can be misalignment of MOA and thus potency where there is in fact similar toxicodynamics (Mackay et al. 2014).

Understanding MOA and chemical mechanisms remains a critical aspect of hazard and risk assessment. The present results, and previous results (Kienzler et al. 2017), show that there is a general lack of concordance in level of specificity and MOA assignment categories across available classification schemes in aquatic ecotoxicology and that each of the schemes can predict a different MOA classification. Also, although the available schemes are all structure‐based, they rely on different computational frameworks for predicting MOA, from structural fragments to theoretical molecular descriptors (Barron et al. 2015). Thus, the development of a unified approach to MOA classification has remained elusive. The present study allowed the establishment of a high‐level consensus across 4 computationally and structurally distinct MOA classification schemes. It is recognized that TEST and ASTER share some similarity in MOA classification approaches, whereas the OASIS and Verhaar schemes differed substantially. The TEST prediction tool was developed using the MOAtox (Barron et al. 2015) database, which incorporated ASTER assignments in the weight‐of‐evidence MOA assignment. However, TEST is computationally distinct because it utilizes a theoretical molecular descriptor approach, whereas ASTER is based on structural alerts, although it is recognized that the MOATox assignments used within TEST rely in part on ASTER (Barron et al. 2015). Collapsing the myriad of specific mechanisms, from acetylcholinesterase inhibition to uncoupling oxidative phosphorylation, into a single category allowed comparison of specifically acting MOAs across classification schemes. Consistent with previous research and independent predictions from each of the 4 schemes, narcosis was the predominant MOA across a diverse group of chemical compounds. This confirms previous findings (Sanderson and Thomsen 2009) and highlights the need for further mechanistic elucidation and understanding of this MOA in particular.

It is acknowledged that uncertainty is inherent when predicting MOA from chemical structure. Yet we believe at the very least the EnviroTox database provides users with a transparent understanding of the variation between MOA classification schemes. In terms of regulatory relevance, information on MOA can be particularly useful when prioritizing organic chemicals where the tolerance for uncertainty is often higher when compared with risk assessment. For example, in the prioritization approach for 640 organic chemicals from Environment and Climate Change Canada (2016), MOA and toxicity ratio rules were responsible for approximately 40% of 195 chemicals being classified as high hazard. Even at a simple binary level, understanding whether a chemical is acting specifically versus nonspecifically allows regulators to infer potential potency. Chemicals acting specifically are generally more potent, often resulting in long‐term sublethal effects (e.g., developmental and reproductive effects). The current MOA approach in EnviroTox cannot address this issue specifically given that aquatic MOA frameworks are predominantly based on acute toxicity; however, indication of a specifically acting MOA may be used as a flag for potential chronic sublethal activity. Knowledge of MOA is therefore a useful indicator for risk assessors during problem formulation stages and can help guide the hazard assessment.

The present study confirms that a higher degree of confidence can be assigned to a narcotic MOA predicted using the structure‐based approaches compared with specifically acting modes. The degree of confidence assigned to the designated MOA becomes very useful for regulatory and nonregulatory applications, where it can be used to accept or reject a predicted MOA according to the tolerated or accepted level of uncertainty. It also becomes evident that the much lower consensus between approaches for specifically acting chemicals is predominantly attributable to lack of sufficient empirical data for these modes and thus lack of sufficient molecular descriptors for structure‐driven MOA approaches. Caution is therefore advisable when interpreting predicted MOA results for specifically acting chemicals in general within and outside of the EnviroTox database. However, we believe that the approach taken in the present study provides a useful binary “narcotic or not” classification for MOA for ecological receptors where a higher reliance on results for a better‐studied specific MOA can be made (e.g., uncoupling of oxidative phosphorylation) in some cases. We suggest to users of EnviroTox that designation of the specific MOA indicated by some approaches such as TEST or ASTER (e.g., neurotoxicity) be regarded as an initial flag to guide a more thorough investigation into the toxicodynamics of a chemical to form a weight of evidence supporting an adverse outcome.

There was also a high proportion of the database (43%) where MOA could not be assigned because of conflicting predictions of MOA (unclassified) or lack of structural coverage for the target chemical. We believe there is insufficient consensus to confidently assign a mode of toxic action. However, the present study and other studies suggest that there is a greater likelihood that an industrial organic chemical has a narcotic MOA. Depending on the context of user needs, it may therefore be useful to initially assign a nonspecific (e.g., narcotic) MOA and conduct further investigations to support or refute this hypothesis using other tools and approaches, as discussed in the present study. This is a practical but nonconservative approach. In the context of chemical prioritization, initially designating unclassified results as having a specific MOA and subsequently seeking coherence with other mechanistic data may be more desirable as a precautionary measure.

Mackay et al. (2014) argue that internal measures of toxicity are far better indicators of chemical potency than external concentrations such as LC50s. One area of future work for the chemicals in EnviroTox could include a comparison of structure‐driven MOA with MOA determined using chemical activity (Mackay et al. 2009), critical body residues (McCarty and Mackay 1993), and the critical membrane concentration (Bittermann and Goss 2017). All of these internal approaches have established internal thresholds for median lethal narcosis (lethal area 50, critical body residue 50, and critical membrane concentration 50, respectively). Calculated tissue residues or activities below the chronic threshold for lethal effects (i.e., using an acute to chronic ratio of 10 applied to the acute median lethal threshold) according to these approaches suggest a specifically acting chemical. Greater confidence with MOA determination can be then assumed where there is concordance of structure and measures of internal toxicity. This type of check on approach coherence was performed for approximately 930 neutral organic chemicals on the Canadian Domestic Substances List by Armitage et al. (2018). The authors compared Environment and Climate Change Canada structure‐derived MOA and critical internal thresholds for median fish lethality data. In summary, 100% concordance across 4 structure and 5 internal threshold approaches was achieved for 53% of the chemicals studied. Far lower concordance (29%) was achieved for specially acting chemicals when all 9 approaches were compared. A much higher concordance was seen when structure MOA and any single internal threshold measure was compared (71–75%) when results for narcotic and specifically acting chemicals were combined. The authors also concluded greater confidence in predicting narcosis and that increased coherence may be achieved with a more thorough curation of the Environment and Climate Change Canada fish median LC50 database used in the comparison. Future efforts could therefore include a similar analysis using the EnviroTox database, given that the empirical toxicity data have undergone curation. It would also be interesting to further evaluate the chemicals classified as narcotics with high acute toxic effects as well as those classified as specific‐acting with low toxicity, to better understand why those unexpected values are occurring (e.g., if this is attributable to a specific MOA that might not be accounted for in MOA approaches, or if it could be explained by a qualitative assessment of the data [duration of test, type of endpoints, etc.]). Future work (and future iterations of the database) could also include the development of tools to better classify the U bin because they represent 43% of the chemical space, as well as other metrics, such as lethal area and critical body residue and tools that can confidently assign specific MOAs (i.e., uncollapse the bins), as they become available. Finally, all current structure‐based MOA approaches rely largely on acute fish lethality data. We encourage research on the applicability domain of these approaches for other aquatic and nonaquatic species.

The present study presents a high‐level consensus across 4 computationally and structurally distinct MOA classification schemes, which provides both a transparent understanding of the variation between MOA classification schemes and an added certainty of the MOA assignment. It provides scientists and regulators with a basic and more reliable understanding of MOA and can aid in the design of additional studies on the prioritization, ranking, and risk assessment of chemicals.

## Supplemental Data

The Supplemental Data are available on the Wiley Online Library at DOI: https://doi.org/10.1002/etc.4531

## Disclaimer

The present study was subjected to review by the US Environmental Protection Agency's (USEPA's) National Health and Environmental Effects Research Laboratory and Environment and Climate Change Canada and approved for publication. Approval does not signify that the contents reflect the views of the USEPA or Environment and Climate Change Canada, nor does mention of trade names or commercial products constitute endorsement or recommendation for use.

## Supporting information

This article contains online‐only Supplemental Data.

Supporting information.

## Data Availability

All information associated with this manuscript are available on the EnviroTox database website at http://www.EnviroToxDatabase.org
